# Screens and scars: SEM analysis of the relationship between childhood trauma, emotion regulation, and social media addiction

**DOI:** 10.3389/fpsyg.2025.1502968

**Published:** 2025-01-24

**Authors:** Nurten Elkin, Ashifa Kariveliparambil Mohammed Ashraf, Oğuzhan Kılınçel, Şenay KılınçeL, Maharshi Ranganathan, Aslıhan Kübra Sakarya, Ayşe Mücella Soydan

**Affiliations:** ^1^Faculty of Health Sciences, Istanbul Gelisim University, Istanbul, Türkiye; ^2^Sakarya Child and Adolescent Psychiatry Institute, Sakarya, Türkiye; ^3^Department of Psychology, Christ University, Bangalore, India

**Keywords:** addiction, social media, childhood trauma, SEM analysis, emotion regulation

## Abstract

**Background:**

Addiction is an increasingly significant global public health concern, affecting individuals across diverse age groups and demographics. With the rapid rise of digital technology, social media addiction has emerged as a growing behavioral issue, impacting mental health, interpersonal relationships, and daily functioning.

**Methods:**

This study employed an online cross-sectional self-report questionnaire, with university students aged 16–35 years as the target population. Data were collected using Google Forms questionnaires, accessible via the university registration system, and sent to the participating students’ smart phones. The data collection instruments included the Social Media Addiction Scale (SMAS), the Childhood Trauma Scale (CTS), and the Difficulty in Emotion Regulation Scale (DERS).

**Results:**

Data from 318 university students were analyzed. The analysis of sociodemographic data revealed a mean participant age of 21.2 years, with 87.3% being female. An analysis of the relationship between social media addiction and childhood trauma revealed that participants with childhood trauma had higher social media addiction. The linear regression model, including childhood traumas and emotion regulation difficulties for social media addiction scores, was statistically significant. A positive correlation was observed between social media addiction and difficulty in emotion regulation.

**Conclusion:**

These findings suggest that individuals who struggle with emotion regulation tend to use social media more frequently. Furthermore, the negative effects of childhood trauma on emotion regulation capabilities during adulthood contribute to the development of social media addiction.

## Introduction

The internet fundamentally changes how we live, think, and interact. In particular, adolescents are more likely than adults to prioritize and cultivate online social connections (Durkee et al., 2016). Social media constitutes a vast platform that facilitates the creation of social indicators that most active social media users fall within the young adult demographic, typically aged between 18 and 29 (Khalaf et al., 2023). Moreover, it was reported that 55–82% of teenagers and young adults regularly engage with social media platforms (Holly et al., 2023).

The entire population, which constitutes 17.6% of the total Internet users, is actively online as of December 2021 (Battista et al., 2021). This aligns with global trends in which media addiction is a growing concern among adolescents (Holly et al., 2023). Characterized by impulsive behavior, this addition can lead to a range of emotional and psychological issues including depression, anxiety, and loneliness. This condition is associated with difficulties in managing emotions and controlling impulses (Yılmazer, 2018). As the use of social media became popular, behavioral, social, and psychological issues leading to addiction became more frequently observed. Social media addiction, characterized by an intense fixation on social media platforms, an excessive craving for their use, and the manifestation of psychological challenges in various domains of life due to prolonged Internet usage, is a behavioral addiction (Henzel and Håkansson, 2021).

Students are becoming more reliant on digital forms of communication and distraction; however, this often leads to social media addiction, which is a rising concern among parents, schools, colleges, and university students (Niaz, 2019). The most notable feature of this addiction is that it relies on the unrestrained and compulsive use of social media, which can create an endemic problem of not only mental health for students but also academic success and social interactions (Khalaf et al., 2023). Adolescents and young adults use social media to stay connected, experience social pressure, and present an online image of themselves (Yule and Canterbury, 1994). This addiction can result in reduced concentration, procrastination, inefficient use of time, increased anxiety, depression, isolation, and poor sleep. If schools and universities can tackle these challenges when it comes to addiction, they may promote more successful strategies that their students can follow.

Social media platforms, such as Facebook, Instagram, and Twitter, have provided young people with the opportunity to travel, express themselves, and maintain friendships (Verzeletti et al., 2016). However, excessive use can result in psychosocial issues including conflicts, declining academic performance, and social isolation (Cataldo et al., 2021). Social media addiction, recognized as a mental health concern, negatively affects cognitive, behavioral, and emotional wellbeing, often leading to communication challenges (Pruthvi et al., 2018). Recent research indicates that approximately 64% of adolescents exhibit moderate-to-high levels of social media addiction.

Emotion regulation, a subset of affect regulation, involves the expression, monitoring, and evaluation of emotions. It encompasses both conscious and unconscious efforts to influence emotional experience (Vrtička and Vuilleumier, 2012). The two most widely used strategies for managing emotions are cognitive reappraisal and suppression. Cognitive reappraisal involves altering the interpretation of negative situations to lessen their emotional impact, whereas suppression aims to inhibit the outward expression of emotions and related behaviors (Gross, 2002). Emotional regulation skills are necessary for people to identify, understand, and accept many emotions; if they do not have these skills, some people may behave impulsively (acting out of rage instead of understanding its roots) or be unable to develop meaningful strategies for regulating their own emotional states (Gross, 1998; Eisenberg et al., 2005).

Emotional stability is affected by age, gender, being left behind, and family economic status (Jangra et al., 2020). According to the Interaction of Person Affect-Cognition-Execution (I-PACE) model (Watters et al., 2024), bio-psychosocial elements, including early developmental experiences, represent vulnerability pathways for addiction (Grolleman et al., 2023). Adverse childhood experiences, such as trauma, may affect brain development in ways that impact memory and self-regulation, thereby increasing the risk of emotional instability (Rose et al., 2019). These traumas, which can range from environmental stressors like snowstorms to physical abuse, play a critical role in shaping brain development and plasticity (Ritblatt et al., 2017). Such experiences are particularly detrimental to younger children, especially those in the critical developmental window of 0–5 years.

Childhood trauma presents high odds for DSM-IV lifetime disorders, and children who have suffered childhood trauma are 1.5 times more likely to develop emotional instability than the general population (Greeson et al., 2013). According to the I-PACE model, a lack of social support can contribute to excessive internet use, which may eventually lead to Internet Addiction (IA). Traumatic childhood experiences can trigger neurobiological changes, making it difficult to express and understand emotions later in life (Cimino and Cerniglia, 2018). Trauma and stressful life events are associated with various adult psychopathologies, including depression, anxiety, post-traumatic stress disorder, and addiction (Heim et al., 2010). Additionally, childhood trauma can increase the risk of Internet addiction, particularly among children raised in dysfunctional families. Although poor social support is associated with childhood trauma, good support may buffer its negative effects, although this has not been consistently demonstrated in all studies.

The relationship between Internet addiction and emotional regulation has been well documented; however, several critical areas remain underexplored. A lack of thorough studies of the specific pathways through which childhood trauma contributes to social media addiction is a problem (Cimino and Cerniglia, 2018). Childhood trauma has a substantial impact on emotional regulation and its subsequent effects on addictive behaviors (Kırcaburun et al., 2020). The I-PACE model sheds light on how biopsychological factors contribute to the emergence of internet addiction. However, more empirical research is required to investigate how early life experiences, particularly trauma, interact with these factors to shape the genesis and course of social media addiction (Cataldo et al., 2021).

The protective role of emotional regulation in mitigating the effects of childhood trauma and social media addiction has been suggested but has not been consistently demonstrated across studies. Research has primarily focused on general social media addiction, with insufficient attention given to its unique characteristics and consequences of social media addiction.

According to previous research, those who have trouble controlling their emotions are more prone to turn to addictive behaviors to suppress or escape uncomfortable feelings. However, it remains unclear what emotional regulation issues play a role in social media addiction. There has not been much research on the interactions among difficulties in emotional regulation, early trauma, and social media addiction. This study aimed to advance the existing literature by investigating the factors contributing to social media addiction and analyzing its connection with childhood experiences and difficulties in emotion regulation. Based on the research purpose, this study attempts to answer the following research question: (i). Does childhood trauma and emotional regulation impact students’ social media addiction behavior? This study surveyed a convenience sample of primary and secondary schools to assess the general prevalence of emotional abuse among adolescents and explore the specific relationship between social media addiction and childhood trauma. This study offers theoretical insights that can aid in preventing internet addiction among adolescents.

## Literature review

Self-Determination Theory (SDT) posits that these three fundamental psychological needs—autonomy, competence, and relatedness—drive human behavior and serve as critical sources of motivation (Wong et al., 2015). According to Deci and Ryan (2008) these needs are universal and essential for optimal psychological functioning. However, when these needs are not adequately met, individuals may seek alternative means to fulfill them, sometimes leading to maladaptive behaviors such as addiction. Although SDT has been extensively applied to contexts such as exercise, its principles are equally relevant to understanding social media addiction (Deci and Ryan, 2008).

Social media platforms, such as exercise, offer environments in which individuals can experience varying degrees of autonomy (the ability to control their interactions), competence (the feeling of effectively navigating and utilizing the platform), and relatedness (the sense of connection with others). When these needs are not satisfied in the real world, individuals may turn to social media as a compensatory mechanism (Altuwairiqi et al., 2019; Khalaf et al., 2023). People who feel stifled in their offline lives, say, by a work environment that is overbearing or a social circle that suppresses their beliefs, may wish to compensate for this with the illusion of freedom and control in an online world. If it feels as though they cannot do anything right in their real life, they gain an easy sense of competence from the instantaneous feedback and validation available through social media (i.e., likes, comments); at least there are things on earth that I am good at doing, which may be the mentality [63].

Social media are built around the community and provide a feeling of connection that is attractive to those who feel alone or isolated offline. However, in the context of meeting relatedness needs, if this is mainly through virtual interactions such as social media communication, this may result in the need to resort exclusively to social media for experiences that are most suitable for creating addictive tendencies (Kuss and Griffiths, 2017). This can create a cycle of addictive social media behavior, in which individuals depend on social media to relieve their deficiencies in psychological needs. Eventually, turning to social media for such needs can gradually grow into an addiction of sort that persists in the long run.

This insight makes SDT an interesting framework for understanding this process, focusing on the imbalances of autonomy, competence, and relatedness that drive individuals toward excessive and compulsive behavior (Koole et al., 2019). This also reinforces the need for an inbuilt motivation to ensure that we continue to have healthy relationships with our activities and social media. People are less likely to engage in addictive behaviors when participating in social media based on intrinsic interest or a self-determined choice (such as connecting with friends for some genuine interaction), which seems to involve fewer people than sending say 1,000 friends requests a week just to get more likes (Lichtenstein et al., 2018). However, at the other end of this continuum, a lack of autonomy, competence, or relatedness combined with extrinsic motivations (e.g., seeking likes or followers) is more likely to fuel social media addiction.

Similar to exercise addiction, motivation is important for the onset and duration/belligerence of social media addiction. For these authors, SDT is crucial for understanding this kind of dynamics (Manchiraju and Sadachar, 2018). Individuals who exercise in healthy ways tend to report higher levels of competence, relatedness, and autonomy than those with low involvement in these types of activities. Likewise, those who use social media more wisely can have these psychological needs better satisfied, thereby mitigating the risk of addiction. In the same vein, however, an essential part of this compulsion may be the result of diminishing autonomy or relatedness, claims that can equally be transferred to social media use. People also naturally desire to fulfill these needs, so when they feel like they have to do these things on social media, this could lead to addiction (Altuwairiqi et al., 2019).

### Childhood trauma and social media addiction

Adverse Childhood Experiences (ACEs) are childhood traumas that are a major public health concern because they have the potential to impact an individual’s physical and mental health throughout their lifetime (Pruthvi et al., 2018). The field commonly uses the term “childhood trauma” more broadly to encompass a range of maltreatment that includes emotional, physical, and sexual abuse, as well as early life neglect. According to Elnour (2017), these traumatic situations can leave lasting marks on children, altering their emotional and cognitive development.

Empirical research has clearly shown that significant disruptions in basic emotional functioning are related to histories of abuse and neglect of child development. For instance, such trauma can severely impair the development of attachment, trust, and emotional self-regulation, all of which are critical for a normal range of emotional expression. Furthermore, impairments in processing, such as difficulty in problem solving and decision-making, also contribute to hurdles that a person may face during daily life. These disruptions do not just end in childhood, but can be apparent throughout adolescence and into adulthood, where they may contribute to the development of different kinds of psychopathologies (Ryan et al., 2017).

The most profound consequence of early life trauma is the development of posttraumatic stress disorder (PTSD). Individuals who had experienced abuse in their childhood are at risk for developing Post Traumatic stress disorder (PTSD) far greater than those who did not (Yurdagül et al., 2021). For example, Shipman et al. found that maltreated children had more severe emotional regulation difficulties than their non-abused peers. This study highlights how trauma compromises children’s ability to regulate and express emotions.

There is a direct relationship between childhood traumatic experiences (CTE) and difficulties in emotion regulation. In other words, early trauma leads to lasting emotional impressions that often render the affected unable to withstand stress or regulate their emotions correctly (Manchiraju and Sadachar, 2018; Yılmazer, 2018). These deficiencies can have long-lasting effects on a person’s life, affecting many things that may or may not make them more prone to addiction.

Theoretical models of addictive behaviors concerning alcohol and drug abuse, gambling, and gaming support an association between experiences in early childhood (Wong et al., 2015). For example, in teenagers over 12 months of age, both males and females separately showed exercise addiction prediction by varying emotion regulation strategies (Lim et al., 2020; Cimino and Cerniglia, 2018). The current study suggests that emotion regulation mediates the relationship between early trauma, childhood environment, and development of addictive behaviors in adult life.

Recent research has focused on the relationship between behavioral addiction (e.g., exercise addiction) and childhood trauma (CT). According to Burgkart et al. (2022). Behavioral addiction is immensely more probable in those who have experienced trauma in their formative youth. This underlines the importance of intervention in these first experiences as a way to prevent this disease from advancing. However, there is little research on the direct association between exercise addiction and CT in the literature published at present (Cain, 2018).

### Emotional regulations and social media addiction

Social media use has become increasingly common in daily life, with many people using these platforms to manage their moods and relieve stress. Social media use is frequently sought as a means of managing emotions and escaping from everyday stressors, much to the function that exercise plays in mood regulation for the general population (Li et al., 2023). However, the psychological processes that distinguish between addicted and healthy social media use patterns can be intricate and closely linked to the ability to control emotions and fulfill basic psychological needs.

According to previous research, those with superior emotion regulation skills are those whose psychological needs are sufficiently satisfied during their formative years, especially during childhood and adolescence (Zaremohzzabieh et al., 2014). Consequently, these abilities have been associated with better results in a number of spheres of life, such as improved general wellbeing and interpersonal interactions. The capacity to successfully manage emotions is essential because failing to do so can have serious negative consequences on physical and mental wellbeing, interpersonal satisfaction, and productivity at work, among other areas. Such difficulties are associated with increased stress levels, heightened vulnerability to mental health disorders, such as anxiety and depression, strained interpersonal connections, reduced productivity, and a general decline in quality of life (Company-Córdoba et al., 2020).

Emotional control is crucial when social media is used. Social media can be a useful tool for mood management, exercise can also be a beneficial technique for emotion regulation (Pruthvi et al., 2018). People may use social media to find upbeat content, receive encouragement from others, or divert their attention from unpleasant emotions. However, when emotional control is lost, using social media can become more than just a constructive coping mechanism; it can also become an addictive habit (Koole et al., 2019).

Maladaptive emotion regulation styles are common among individuals with risk factors of social media addiction. People with a limited self-regulation tendency to cope with emotional unrest spend longer hours on social media (Zaini et al., 2023). Such behavior can spark a negative feedback loop, in which the individual becomes more reliant on social media for emotion regulation and an eventual progression to compulsive use patterns. These maladaptive coping mechanisms lead to dependency, which is expressed in social media (Altuwairiqi et al., 2019) or physical activities such as excessive exercise.

In the cognitive-behavioral paradigm, it is important to highlight the role of emotional regulation in sustaining behaviors, such as addiction to exercise (Li et al., 2023). This model is different from most other models, which focus predominantly on the behavioral or cognitive elements of addiction, as it underscores the emotional dimension of addiction. This concept is also a new theory that people who have difficulty with emotional regulation, such as social media, are at a risk of addiction (Zhao et al., 2022). Under this framework, the idea of using social media to manage or escape emotional distress is another missed opportunity for clear additional evidence in support of negative coping behavior.

**Figure 1:**
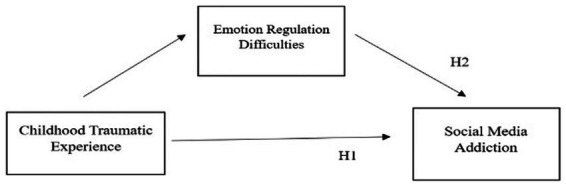
Visual representation of hypothetical model.

Research has shown that individuals with lower emotion regulation capacities are most likely to use addictive behaviors in response to their emotions (Cain, 2018). This idea also applies to social media addiction: individuals who have trouble controlling their emotions may find that using social media helps them avoid confronting their emotions, which can lead to a vicious cycle of compulsive use. Social media addiction can become ingrained over time, making it difficult for users to cut ties with the platform, even when it severely impacts other areas of their lives (Deniz and Deniz, 2015). This research aims to enhance the understanding of social media addiction and its associated factors, as well as develop prevention strategies for social media addiction, based on these hypotheses. Figure 1 illustrates the theoretical model used in this study.

*H1*: Students who have experienced childhood trauma must be addicted to social media.

*H2*: Students with emotional regulation difficulties must be addicted to social media.

*H3*: Difficulties in emotion regulation mediate the relationship between childhood trauma and social media addiction.

Figure 1 illustrates the theoretical model used in this study.

## Materials and methods

### Participants

The study sample includes individuals aged 16–35 who have been using social media frequently (more than 2 h a day) over the past month. Participants were recruited through social media platforms specifically targeting users of Instagram, Facebook, and YouTube, which are highly popular among students in the research area. The criteria for participant selection were: (i) between the ages of 16 and 35, (ii) having used at least one social media platform in the past 6 months, and (iii) having an average daily social media usage of more than 2 h.

**Figure 2:**
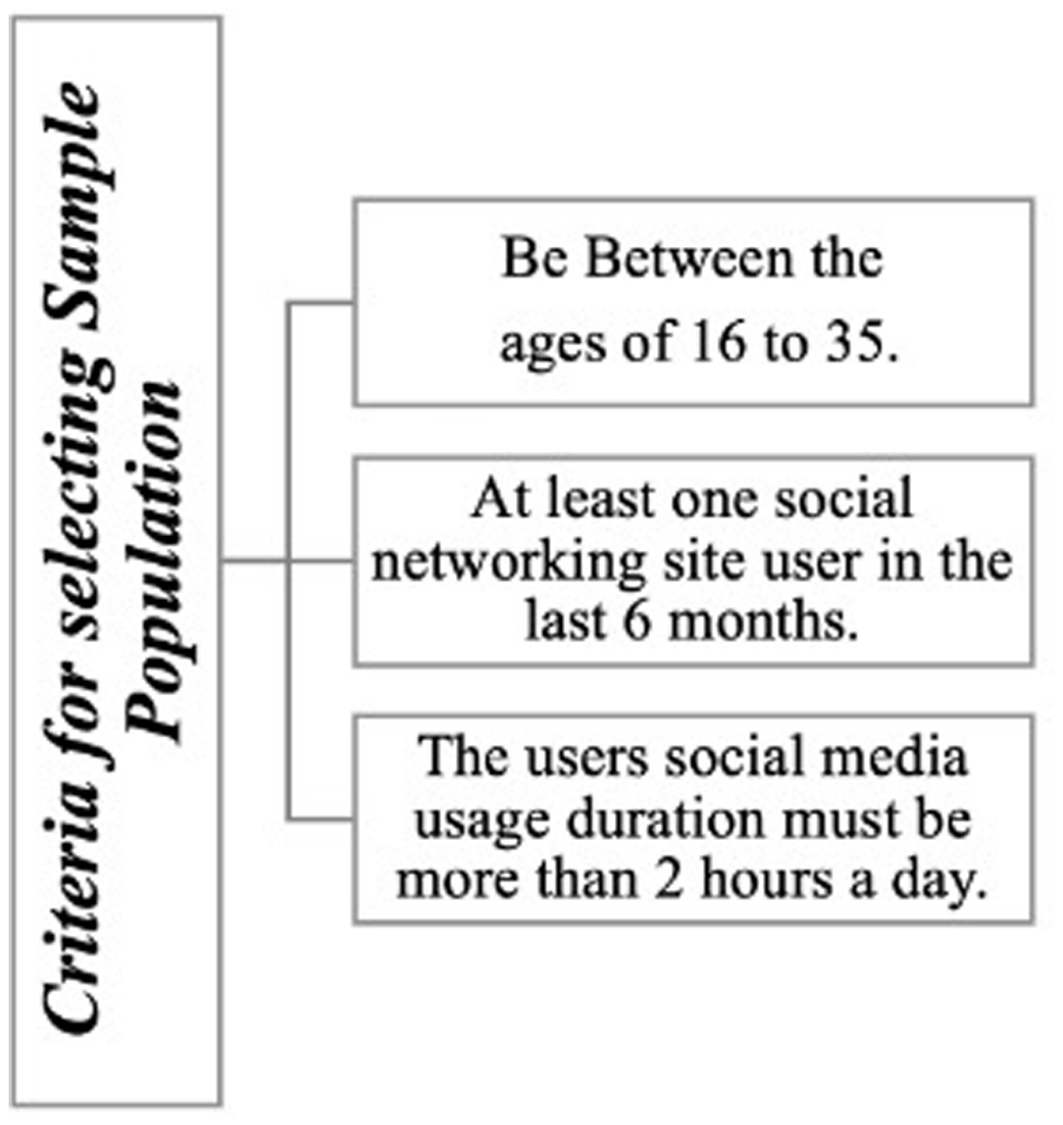
Criteria for selecting sample population.

Convenience sampling was employed to collect the participants. This study used an online cross-sectional self-report questionnaire targeting university students. Data collection was facilitated through a Google Forms questionnaire accessed via the university registration system and sent to the smartphones of the university participants. In total, 350 responses were obtained. After completing the screening process, 318 valid data points were used for data analysis. This research project received ethical approval from our institutional ethics review committee (04.11.2022/2022-16). The criteria for the sample are shown in Figure 2.

### Measurements

The researcher used the Social Media Addiction Scale-Adult Form (SMAS-AF) to assess pathological social media use. This scale consists of 20 items and employs a five-point scoring system, with responses ranging from one (“not at all”) to 5 (“always”). The total score ranged from 20 to 100. Participants scoring below 50 were classified as not addicted to social media, whereas those scoring above 50 were considered addicted. The scale was developed by Şahin and Yagci (2017) (Sheldon et al., 2020). The scale demonstrates strong internal consistency, with a reported Cronbach’s alpha coefficient typically above 0.85, indicating high reliability.

The Childhood Trauma Questionnaire Short Form (CTE) was used to assess the traumatic experiences that occurred throughout childhood. The CTE, which is derived from consists of 25 questions divided into five subscales: emotional abuse, physical abuse, sexual abuse, emotional neglect, and physical neglect. Each subscale consists of five items. A five-point rating system was used to categorize responses: never, occasionally, sometimes, often, and always. The handbook states that each subscale has a point range of 5–25, whereas the scale’s overall score varies from 25 to 125 (Sar et al., 2012). The scale demonstrates excellent internal consistency, with Cronbach’s alpha values typically exceeding 0.80 for both the overall scale and individual subscales. The Turkish adaptation of Emotional Regulation Skills (DERS) scale was undertaken by Ruganci and Gencoz (2010) was used to assess individuals’ difficulties with emotion regulation. This scale features six subdimensions: awareness, clarity, non-acceptance, strategy, impulse, and goals. Thirty-two items total, each using a 5-point Likert scale for rating. Greater difficulty in regulating emotions is indicated by higher DERS scores. The Turkish version has shown good internal consistency and test–retest reliability, with Cronbach’s alpha values above 0.85 for the total scale.

### Statistical analysis

SPSS software (version 22.0, SPSS Inc., Chicago, IL, USA) was used for statistical analysis. Before conducting the planned analyses, they assessed the normality of the variables and their subdimensions. The dataset was first examined for outliers and for normality. Data from 32 students who did not meet parametric conditions were excluded. Normal distribution conformity was evaluated using the Kolmogorov–Smirnov and Shapiro–Wilk tests, confirming Mardia’s assumptions of multivariate normality (*p* > 0.05). Consequently, the analysis was based on data from 318 participants. Descriptive statistics are reported as means, frequencies, and percentages. Pearson’s correlation test was used for correlation analyses, and a structural equation model (SEM) was employed for modelling, with the significance level set at *p* < 0.05.

## Results

Analysis of sociodemographic data (Table 1) revealed a sample of young adult females from urban areas with moderate income levels, with an average age of 21.2 years. Among all participants, 87.3% were women. Our analysis revealed that 61.8% of the participants grew up in urban centers and 88.8% described their families’ financial income as moderate.

**Table 1:** General sociodemographic data of social media addicted users.

Demographic profile	Categories	Total sample (*N* = 318) (%)
Age (*x* ± *s*)	16–35	21.2 ± 1.35
Gender	Male	40 (12.7%)
	Female	278 (87.3%)
Area	Rural	121 (38.2%)
	Urban	197 (61.8%)
Family Income	Better	24 (7.5%)
	Moderate	282 (88.8%)
	Poor	12 (3.7%)
Most used social media	Instagram	268 (84.4%)
	Facebook	11 (3.5%)
	YouTube	38 (11.9)
Preferrable engagement actives	Social activities	66 (20.7%)
	Entertainment activities	61 (19.1%)
	Cultural activities	57 (17.9%)
	None	134 (42.2%)
Response on involvement in emotional relationship	Yes	154 (48.3%)
	No	164 (51.7%)

The study also revealed that a significant proportion of participants did not prefer engaging in specific activities, suggesting a tendency toward passive engagement or a lack of interest in traditional activities. Social activities were slightly more popular than entertainment and cultural activities. 20.7 Of the participants, 20.7% were frequently engaged in social activities and 48.2% reported being in an emotional relationship. Emotional relationships were a relevant but not overwhelmingly dominant aspect of life for the participants. Instagram was the most commonly used social media platform, with 84.5% of participants spending most of their time on it. These demographic data provide a foundation for understanding social media usage patterns and behaviors in this group, with a strong indication of Instagram’s central role.

Table 2 presents the validity and reliability of each construct. The Childhood Traumatic Experience construct has good internal consistency, with a Cronbach’s Alpha (CA) value of 0.822, indicating good internal consistency. The constructs probed by the items were detected in a similar manner. A good composite reliability score (CR > 0.7) was observed, which was widely accepted in previous studies (Kim et al., 2023). An Average Variance Extracted (AVE) value of 0.316 indicates that less than 50% of the variance from the construct is less likely to be explained by items that represent such actions. The emotion regulation difficulty construct had a high CA, suggesting excellent internal consistency. Items measuring this construct were consistently evaluated. Composite reliability (CR) is also excellent, and the AVE value is close to 0.5; which means that almost half of the variance is due to the construct itself, not random measurement error. Social Media Addiction has high CA, which is indicative of internal consistency. Cronbach’s alpha and AVE for all items show this construct to be measured reliably (*α* = 0.731 and AVE = 0.71), indicating that a high percentage of the variance is explained by the construct itself with very little measurement error, as indicated in Table 2. Together, these constructs present good reliability and valid representation, reflecting a clearly defined and measured construct. As improperly translated items of the intended constructs could be drawn throughout this measurement, it was not appropriate to analyze the reliability of these measures. However, the somewhat small AVE for CTE indicated that some items may need improvement to increase their representational nature, which exhibited strong reliability, suggesting that the items effectively measured the intended constructs. However, the relatively low AVE for CTE suggests that some items may require refinement to enhance their representativeness.

**Table 2:** Constructs validity and reliability.

Constructs	Cronbach’s Alpha (CA)	Composite Reliability (CR)	Average-Variance-Extracted (AVE)
Childhood traumatic experience	0.822	0.737	0.316
Difficulties in emotion regulations	0.917	0.805	0.489
Social media addiction	0.801	0.731	0.71

In order to identify the discriminant validity (Table 3) the study examines the correlations between three constructs: Childhood Traumatic Experience (CTE), Difficulties in Emotion Regulation (DER), and Social Media Addiction (SMA). The square roots of the Average Variance Extracted (AVE) for each construct were interpreted using a Fornell-Larcker criterion table. The results showed that CTE was a distinct construct with a moderate relationship with Difficulties in Emotion Regulation (0.412) and a weaker relationship with Social Media Addiction (0.217). Difficulties in Emotion Regulation were moderately well represented by its indicators, with a moderate positive correlation between DER and CTE and a moderate positive correlation between DER and SMA (0.406). Social Media Addiction is moderately distinct as a construct but shows a moderate relationship with Difficulties in Emotion Regulation and a weaker relationship with Childhood Traumatic Experience. The square roots of the AVE values for each construct were greater than their correlations with other constructs, indicating good discriminant validity.

**Table 3:** Discriminant validity.

Constructs	CTE	DER	SMA
Childhood traumatic experience	**0.969**		
Difficulties in emotion regulations	0.412	**0.793**	
Social media addiction	0.217	0.406	**0.742**

Bold values represent the square root of the Average Variance Extracted (AVE).

The descriptive analysis is presented in Table 4. The study revealed that, the Social Media Addiction Scale (SMA) revealed a total mean score of 16.996, with a standard deviation of 1.583. Among the subscales, the highest mean score was observed in the (VP) with a mean of 4.464 (SD = 0.727), indicating that participants commonly experience problems related to their social media usage. This was followed by Virtual Communication (VC), where the mean was 4.389 (SD = 0.703), suggesting that participants frequently engaged in communication through social media platforms. Virtual Information (VI) had a mean of 4.163 (SD = 0.819), reflecting the participants’ reliance on social media for gathering information. Finally, Virtual Tolerance (VT) had a mean of 3.964 (SD = 1.027), showing moderate tolerance toward prolonged social media use. These results show that the entire sample was collectively more addicted to social media. The results of the Childhood Trauma Experience (CTE) scale demonstrated that participants reported considerable levels of trauma, with a total mean score of 21.272 (SD = 2.761). Physical Neglect (PN) had the highest average at 4.337 (SD = 0.657), followed by Emotional Neglect (EN), with an M of 4.266 (SD = 0.771). Subscales PA and EA each had a mean score of 4.298 (SD = 0.995; SD = 0.950). Sexual Abuse (SA) showed a similarly high mean score of 4.274 (SD = 1.049). These results point to the high rate of childhood trauma in this group of participants. Descriptive analysis of the Difficulties in Emotion Regulation Scale (DERS) demonstrated a total mean score of 28.08 (SD = 2.891), indicating that participants experienced significant difficulty in regulating their emotions. The participants also struggled to understand their emotions (awareness = 4.155, SD = 1.000; clarity = 4.194, SD = 0.914). The non-acceptance subscale had a mean score of 4.159 (SD = 0.944), indicating a low acceptance of emotional experiences. The impulse subscale had a mean score of 4.167 (SD = 0.881), showing impulsive behaviors when experiencing emotional situations. Strategies and Goals had mean scores of 4.119 (SD = 0.848) and 4.091 (SD = 0.904) respectively, giving an indication that setting and achieving goals was challenging when emotionally distressed The high levels of social media addiction and emotional regulation deficits in the sample revealed above indicate that participant had extensive histories with adverse childhood experiences.

**Table 4:** Descriptive analysis.

Latent variables			Mean	SD	Minimum	Maximum
Social Media Addiction Scale (SMA)	Virtual tolerance	VT	3.964	1.027	1.000	5.000
	Virtual communication	VC	4.389	0.703	1.000	5.000
	Virtual problem	VP	4.464	0.727	1.000	5.000
	Virtual information	VI	4.163	0.819	1.000	5.000
	Total	SMA	16.996	1.583	4.000	20.00
Childhood Trauma Experience (CTE)	Physical neglect	PN	4.337	0.657	1.000	5.000
	Emotional neglect	EN	4.266	0.771	1.000	5.000
	Physical abuse	PA	4.298	0.995	1.000	5.000
	Emotional abuse	EA	4.298	0.950	1.000	5.000
	Sexual abuse	SA	4.274	1.049	1.000	5.000
	Total	CTE	21.272	2.761	5.000	25.00
Difficulties in Emotion Regulation Scale (DERS)	Awareness	DERS1	4.155	1.000	1.000	5.000
	Clarity	DERS2	4.194	0.914	1.000	5.000
	Non-acceptance	DERS3	4.159	0.944	1.000	5.000
	Strategies	DERS4	4.119	0.848	1.000	5.000
	Impulse	DERS5	4.167	0.881	1.000	5.000
	Goals	DERS6	4.091	0.904	1.000	5.000
	Total	DERS	28.08	2.891	6.000	30.00

The correlation analysis presented in Table 5 indicates significant relationships between the various latent variables measured by the Social Media Addiction Scale (SMA), Childhood Trauma Experience (CTE), and the Difficulties in Emotion Regulation Scale (DERS). Within the SMA subscales, Virtual Tolerance (VT) and Virtual Communication (VC) were highly correlated (*r* = 0.684), suggesting that participants who tolerate spending long periods online also engage heavily in communication through social media. A similarly high correlation was observed between Virtual Problem (VP) and Virtual Tolerance (VT) (*r* = 0.583), indicating that those experiencing issues related to excessive social media use were more tolerant of the time they spent online. The overall SMA score was highly correlated with all its subscales, especially Virtual Tolerance (*r* = 0.928) and Virtual Communication (*r* = 0.892), highlighting that these components are central to participants’ addiction to social media. For the Childhood Trauma Experience (CTE) subscales, correlations between Physical Neglect (PN) and SMA subscales were generally weak (*r* = 0.079 for SMA total), whereas Emotional Neglect (EN) showed a stronger relationship with SMA (*r* = 0.295). The strongest relationship was found between Physical Abuse (PA) and total childhood trauma (CTE) (*r* = 0.802), indicating that physical abuse was a major contributing factor to the overall trauma experienced by participants. Regarding Difficulties in Emotion Regulation (DERS), Non-Acceptance (DERS3) showed moderate correlation with both SMA (*r* = 0.398) and CTE (*r* = 0.633), suggesting that difficulties in accepting emotions may be related to both childhood trauma and higher social media addiction. Additionally, Impulse (DERS5) was moderately correlated with CTE (*r* = 0.688) and SMA (*r* = 0.305), indicating that impulse control problems may mediate the relationship between trauma and addictive behavior. These correlations highlight the interrelatedness of childhood trauma, emotional regulation difficulties, and social media addiction with certain forms of trauma such as physical abuse, showing stronger relationships with addiction outcomes.

**Table 5:** Correlation coefficient of subscales.

Sl	Variables	1	2	3	4	5	6	7	8	9	10	11	12	13	14	15	15	16
1	VT	1																
2	VC	0.684	1															
3	VP	0.583	0.432	1														
4	VI	0.490	0.578	0.483	1													
4	SMA	0.928	0.892	0.769	0.643	1												
6	PN	0.076	0.069	0.089	0.134	0.079	1											
6	EN	0.278	0.263	0.447	0.278	0,295	0.005	1										
7	PA	0.376	0.330	0.364	0.321	0.386	0.012	0.708	1									
8	EA	0.017	0.039	0.132	0.031	0.030	0.445	0.004	0.013	1								
9	SA	0.083	0.063	0.081	0.792	0.080	0.162	0.218	0.191	0.011	1							
10	CTE	0.330	0.302	0.347	0.387	0.345	0.408	0.791	0.802	0.377	0.456	1						
11	DERS1	−0.123	−0.110	−0.163	0.110	−0.127	0.192	0.106	0.103	0.237	−0.040	0.190	1					
12	DERS2	0.191	0.158	0.192	0.183	0.191	0.050	0.655	0.578	0.109	0.163	0.614	0.238	1				
13	DERS3	0.379	0.350	0.323	0.451	0.398	0.045	0.633	0.808	0.002	0.193	0.689	−0.137	0.399	1			
14	DERS4	0.343	0.345	0.332	0.386	0.375	0.005	0.819	0.789	−0.004	0.183	0.744	0.015	0.491	0.726	1		
15	DERS5	0.305	0.299	0.278	0.131	0.329	−0.028	0.811	0.688	−0.065	0.169	0.667	−0.040	0.504	0.656	0.807	1	
16	DERS6	0.226	0.197	0.195	0.341	0.231	−0.020	0.717	0.579	−0.039	0.220	0.598	0.122	0.382	0.475	0.688	0.550	1
16	DERS	0.333	0.317	0.151	0.349	0.354	0.050	0.883	0.855	0.040	0.210	0.829	0.223	0.641	0.787	0.921	0.847	0.761

### SEM analysis

The results of the Model Fit Indices (Table 6) suggest that the model fit the data well. The Chi-square value (*χ*^2^ = 169.45) with 102 degrees of freedom suggested an acceptable fit, especially when combined with other indices. The Comparative Fit Index (CFI) was 0.972, and the Tucker-Lewis Index (TLI) was 0.961, both of which indicate excellent fit (values above 0.95 are considered excellent). The Root Mean Square Error of Approximation (RMSEA) was 0.03, also indicating an excellent fit, as values below 0.05 are considered optimal. Finally, the Standardized Root Mean Square Residual (SRMR) was 0.04, further supporting that the model represented the data well. These fit indices suggest the robustness of the SEM model in explaining the relationship between the CTE, DERS, and SMA.

**Table 6:** Model fit indices.

Index	Values	Remarks
Chi-square (*χ*^2^)	169.45	Indicates an acceptable model fit, especially when combined with other indices.
Degrees of Freedom (df)	102	A reasonable df value; higher degrees of freedom suggest a well-specified model.
AIC (Akaike Information Criterion)	6,898.89	The AIC value indicates good fit relative to other models. Lower is better.
BIC (Bayesian Information Criterion)	6,166.211	The BIC value is used for model comparison. A lower BIC suggests better fit.
CFI (Comparative Fit Index)	0.972	Excellent fit. A value above 0.95 indicates the model fits the data well.
TLI (Tucker-Lewis Index)	0.961	Excellent fit. A value above 0.95 shows strong model performance.
RMSEA (Root Mean Square Error of Approximation)	0.03	Excellent fit. A value below 0.05 is considered excellent.
SRMR (Standardized Root Mean Square Residual)	0.04	Excellent fit. A value below 0.05 indicates minimal differences between observed and predicted values.
Chi-Square (*χ*^2^)/df Ratio	1.66	Excellent fit. A ratio under 2 is indicative of a strong model fit.

The Residual Variance (Table 7) shows the variance that remains unexplained for each indicator within the latent variables. For the Childhood Trauma Experience (CTE) construct, indicators like Sexual Abuse (SA) and Physical Abuse (PA) have relatively higher residual variances, with estimates of 0.75 and 0.71, respectively, suggesting that a significant amount of variance is not explained by the latent variable. For the Social Media Addiction (SMA) construct, Virtual Problem (VP) and Virtual Communication (VC) show residual variances of 0.53 and 0.57, respectively, indicating that while the model accounts for a large part of the variance, there is still some unexplained variability. Difficulties in Emotion Regulation (DERS) indicators, such as impulse (DERS5) and strategies (DERS4), also show moderate residuals, which suggests room for improvement in measuring these latent variables and exploring other potential factors that influence these constructs.

**Table 7:** Residual values.

Latent variable	Indicator	Estimate	Std. Error	*z*-values	*p*	95% Confidence Level		Standardized values
						Lower	Upper	
CTE	SA	0.75	0.083	9.01	<0.001	0.7	0.8	0.75
	EA	0.62	0.086	7.13	<0.001	0.58	0.66	0.62
	PA	0.71	0.082	8.57	<0.001	0.66	0.76	0.71
	EN	0.68	0.010	6.82	<0.001	0.65	0.71	0.68
	PN	0.63	0.089	7.04	<0.001	0.61	0.65	0.63
SMA	VI	0.67	0.083	8.03	<0.001	0.63	0.71	0.67
	VP	0.53	0.090	5.91	<0.001	0.49	0.57	0.53
	VC	0.57	0.087	6.52	<0.001	0.53	0.61	0.57
	VT	0.83	0.130	6.01	<0.001	0.74	0.82	0.83
DERS	DERS1	0.78	0.104	5.58	<0.001	0.55	0.61	0.78
	DERS2	0.55	0.098	5.91	<0.001	0.55	0.61	0.55
	DERS3	0.58	0.104	5.58	<0.001	0.55	0.61	0.58
	DERS4	0.61	0.142	4.29	<0.001	0.58	0.64	0.61
	DERS5	0.52	0.106	4.92	<0.001	0.49	0.55	0.52
	DERS6	0.68	0.115	5.89	<0.001	0.64	0.72	0.68

**Figure 3:**
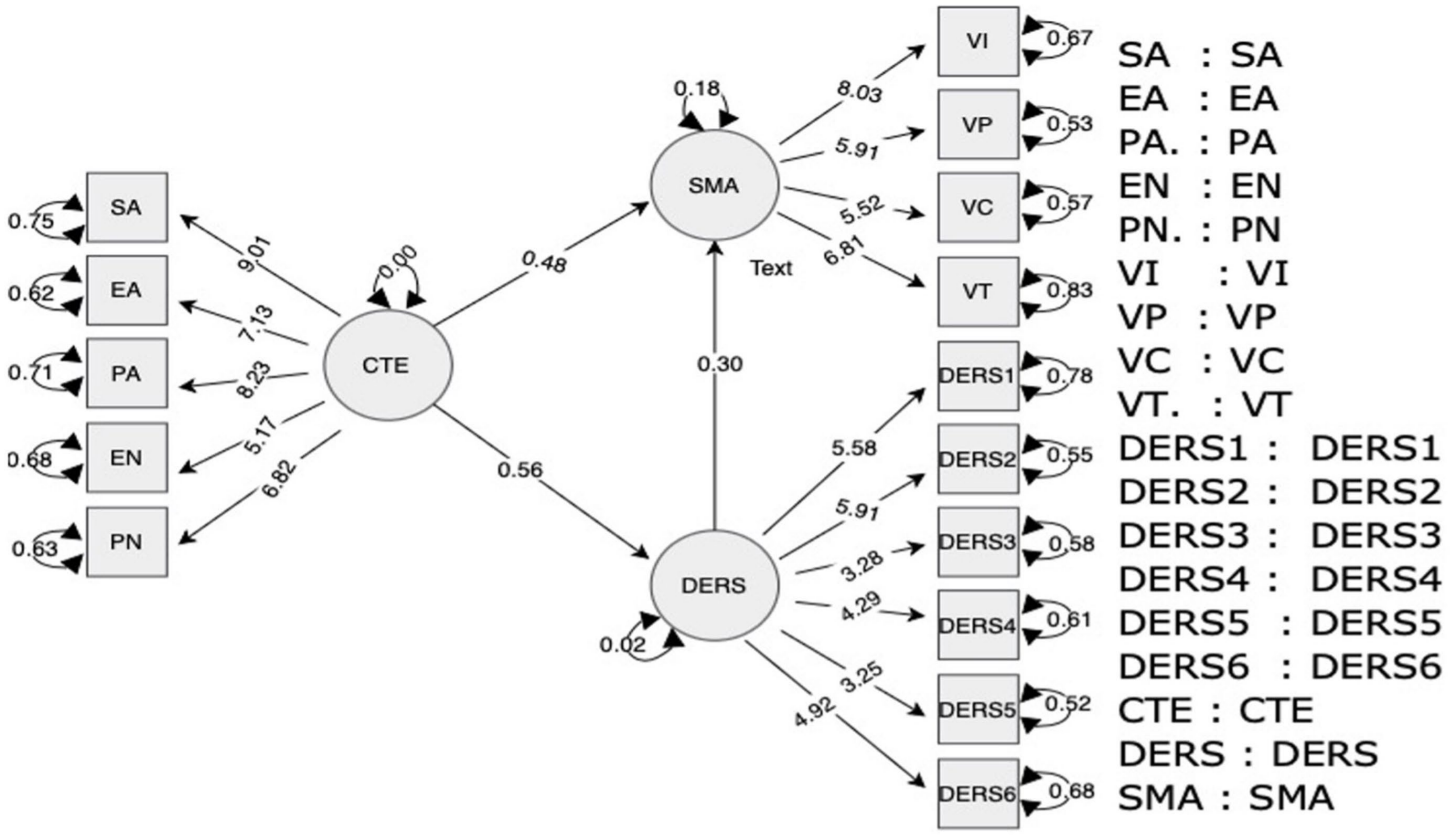
Structural model.

The Path Diagram (Figure 3) visually represents the structural relationships between the constructs. CTE had a direct positive effect on both DERS and SMA, whereas DERS directly influenced SMA. The residuals for the individual items (e.g., SA, EA, and PA) show how much of the variance remains unexplained, aligning with the results from the residual variance table.

The Regression Coefficients (see Table 8) represent the paths between the key constructs. The direct effect of Childhood Trauma Experience (CTE) on Social Media Addiction (SMA) was significant (estimate = 0.48, *p* < 0.001), supporting Hypothesis 1. This finding indicates that higher levels of childhood trauma lead to higher levels of social media addiction. Hypothesis 2, which proposed a path from CTE to Difficulties in Emotion Regulation (DERS), was also supported (estimate = 0.56, *p* < 0.001), indicating that childhood trauma significantly affected emotional regulation difficulties. Finally, Hypothesis 3 was confirmed, with the DERS acting as a significant predictor of SMA (estimate = 0.30, *p* < 0.001), suggesting that emotion regulation difficulties mediate the relationship between childhood trauma and social media addiction.

**Table 8:** Regression coefficient.

Hypothesis	Path	Estimate	Std. Error	*z*-value	*p*	95% Confidence Interval		Standardized values
						Lower	Upper	
H1	CTE → SMA	0.48	0.08	6.00	<0.001	0.32	0.64	0.48
H2	CTE → DERS	0.56	0.56	5.60	<0.001	0.36	0.76	0.56
H3	DERS → SMA	0.30	0.09	3.33	<0.001	0.12	0.48	0.30

## Discussion

This study recruited university students to explore the relationship between social media addiction, childhood experiences, and difficulties with emotion regulation. The study sample had a higher proportion of female participants, suggesting that women use social media platforms more extensively than men. Studies of the association between gender and social media addiction have yielded inconsistent results. However, some studies say that men have a higher proclivity toward social media addiction, while others claim that it is women. However, Köse and Doğan (2019) did not observe gender differences in social media (Köse and Doğan, 2019). Childhood trauma is also significantly correlated with social media addiction, according to the study.

Results from the Social Media Addiction Scale (SMA) revealed that the Virtual Problems (VP) and Virtual Communication (VC) subscales both indicated signs of significant addiction. This indicates that some respondents may find it difficult to manage their online communication, potentially resulting in unfavorable consequences, such as those in other aspects of life. This finding is in line with a previous study on how social media addiction predicts difficulties in balancing offline life with online usage (Steers et al., 2016).

In the path analysis, exposure to physical abuse and the risk of social media addiction showed an important correlation. This finding is consistent with the association described in multiple studies (Figure 2). The results of these studies point to the importance of childhood experiences, specifically early exposure to trauma, in shaping the lives of adults. For example, Chegeni et al. (2023) indicated that individuals who experienced neglect and abuse in childhood are at a higher risk of Internet addiction (Chegeni et al., 2023).

Their report also stressed that people who have suffered neglect appeared to have more alexithymia; that is, they had a harder time identifying and valuing their own emotions. Additionally, a study of undergraduate students reported that undergraduates with social media addiction had suffered from traumatic events in the past more frequently than those without social media addiction (Cain, 2018). Studies with adolescents have suggested that exposure to childhood trauma increases the risk of substance use disorders as well as social media addiction in adulthood (Pruthvi et al., 2018). The participants were highly traumatized (Childhood Trauma Experience [CTE] results were all above trauma cut-offs), particularly in terms of Physical and Emotional Neglect. Another explanation may be provided by studies such as Chegeni et al. (2023), where emotional disturbances were linked to childhood neglect, which increased the risk of social media addiction due to the inability to cope with emotions (Chegeni et al., 2023).

This study found that individuals who had encountered physical violence had a markedly increased risk of social media addiction. This result significantly contributes to the literature. Consistent with the literature, we found a positive correlation between social media addiction and emotional dysregulation difficulties. Nazlıgül et al. (2022) found that those with impairments in emotion regulation were more likely to suffer from smartphone addiction, supporting this argument (Nazlıgül et al., 2022). In contrast, greater smartphone addiction was correlated with a worse ability to regulate emotions. In line with this finding, individuals with emotion regulation difficulties might be inclined toward addictive behaviors to escape their problems and alleviate their negative affect.

Participants had substantial difficulty controlling and modulating their emotions according to the Difficulties in Emotion Regulation Scale (DERS). The highest total scores were obtained for Impulse Control and Non-acceptance, which were the components on which participants reported more difficulties in controlling their impulses or accepting their emotional states. This is consistent with the premise that emotion regulation problems underlie the relationship between childhood trauma and social media addiction as people may go to social media to cope with or reduce negative emotions (Hou et al., 2019).

Similarly, social media addiction is also described as functioning similarly to behavioral addiction, and it has been suggested that individuals with this type of addiction could suppress or avoid emotions rather than genuinely appraise them (characterized). People showing pathological social media use found it difficult to identify, articulate, or control their feelings. Moreover, 318 college students showed a significantly negative relationship between Facebook addiction and emotional intelligence (Zhou, 2021). The issues in this group were related to an inability to accept emotional reactions, use of inadequate regulatory strategies, reduced impulse control, and difficulty using goal-directed behavior. One of the major takeaways of our research is that high emotional awareness is a protective factor against social media addiction. Emotion recognition and understanding are the foundations of dealing with earlier, negative emotional experiences (Zaremohzzabieh et al., 2014), and emotional awareness was found to be one of the most important components of overall psychological health.

This relationship was further verified using correlation analysis. This indicates that using social media for both Virtual Tolerance (VT) and Elongated Usage VT with regard to either tiredness makes it such that these two unique instances in social media addiction are ultimately highly correlated, suggesting a biological basis in which the need for long periods of screen time is driven by the ease of communication. The close relationship between VP and VT may be explained by society or individuals being affected by social media addiction (Steers et al., 2016).

This study found a strong positive relationship between childhood trauma and emotional regulation difficulties with social media addiction. Based on our results and those of this study, we conclude that childhood trauma has been significantly correlated as a principal predictor of addiction to social media among addicted users who are prone to being affected by newer cases in terms of using various addictive online apps. This highlights the lasting mental effects of trauma, in which people may adopt unhealthy coping strategies. The study also revealed a moderate positive link between childhood trauma and disruption in emotion regulation, implying that individuals with a traumatic past may have trouble regulating their emotions. However, the relatively modest strength of this relationship suggests that other factors, including potential genetic predisposition and social support or later life experiences, are likely to also play a role.

SEM analysis also provides significant insights into these relationships. The model fit indices indicated that the hypothesized model fit the data well, with excellent CFI (0.972), TLI (0.961), and RMSEA (0.03). This supports the robustness of the model in explaining how childhood trauma, emotion regulation difficulties, and social media addiction interact.

Difficulties in emotion regulation partially mediate the relationship between childhood trauma and social media addiction. This suggests that while emotion regulation challenges contribute to the pathway from trauma to addiction, the direct impact of trauma is still a more significant factor. This highlights the importance of directly addressing trauma in therapy rather than focusing solely on symptoms. The findings of this study have important implications in the treatment and prevention of social media addiction. Prevention efforts should emphasize early intervention and support for at-risk children, whereas treatment approaches should be comprehensive, addressing both trauma and difficulties in emotion regulation. This suggests that the most effective approach to recovery may be trauma-focused therapies combined with interventions aimed at improving emotional regulation. Similarly, those who have experienced emotional neglect or physical abuse are likely to struggle with the ability to identify and regulate emotions, control impulses in response to negative stimuli, perform tasks effectively while experiencing negative emotions, or comfort themselves following a distressing situation.

The mediation effect of DERS adds that people with childhood trauma have a higher likelihood of social media use for experiencing emotion regulation problems, which resolves their own psychological risks. Such evidence indicates that interventions would promote emotional regulation to reverse the effects of trauma on addiction behaviors (Wu et al., 2015).

Research on abuse is underway to reinforce the importance of our results. In particular, victims of emotional abuse are prone to Internet addiction. As previously demonstrated, the occurrence of physical abuse in childhood is associated with a high frequency of poor self-esteem and escape from the Internet (Ballarotto et al., 2018). Another study found significant emotion dysregulation among male NIBD cases, specifically in a subgroup of male subjects with alcohol and opiate addiction (Kim et al., 2023), from the same prior study that also reported childhood emotional neglect. Moreover, they had difficulty applying more effective strategies to cope with negative affective states and showed a reduction in goal-directed behavior. Various studies have investigated the trend of emotional regulation on a number of other addiction theoretical chemical dependency substances and tweed propagating this model in order to account only for those with respect to behavior addiction as an overarching impulse—control trait (Noël et al., 2013).

Taking our findings and putting them together with the existing literature indicates that people with less emotion regulation ability respond to their emotions differently. This personal drive often stems from struggles with impulsivity and underdeveloped impulse control mechanisms that disable the process of being able to simply interacting or finding divine solace in any form of social media (Yılmazer, 2018). This is compounded by the persistent effect of early childhood trauma on impaired emotion regulation in later life, which lays the groundwork for addiction to social media. Proactivity in protecting children from suffering during childhood and offering them strong emotional and physical care would lead to a decrease in numerous addictions, and will be especially beneficial toward countering social media addiction [63].

This allowed them to learn more applied emotion regulation skills in real-life. It is also worth mentioning that prevention and early intervention approaches—designed to stop addiction before it starts—are key band aids in the fight against it. Therefore, they need inclusive education regarding addiction and psychological counseling, in addition to more enlightening measures for university students. Human social workers will need to know about new forms of addiction to use their potential for further education, and other educational programs can reduce the harmful impact of addictive behavior. However, we need courses on addiction prevention and intervention at universities. Public awareness campaigns coupled with policy interventions that target childhood trauma and other factors correlated with social media addiction can help identify individuals at risk of compulsive usage soon enough to offer group assistance in mitigating the use of socio-digital platforms as palliative care.

## Implications

The research also helps to better understand the complicated link between trauma in childhood and excess emotion connected with addiction to social networks among students. The latter suggests that negative early life experiences can have a lasting influence on students’ regulation of emotions, potentially culminating in the development of maladaptive coping mechanisms. Emotion dysregulation is also considered an important characteristic in prevention and intervention, because our study shows a significant mediating effect between emotion dysregulation and addiction, and we need to interpret that addictive behaviors may be coping strategies for students with difficulty in emotional control. This has important implications for the explanatory mechanism of SNS addiction (SNSA) and suggests that both trauma and emotion regulation theories need to be incorporated into its addiction model, which may strengthen our understanding of etiological pathways and thereby facilitate the development of more effective prevention/intervention strategies.

### Theoretical implication

The relationship between social media addiction, childhood trauma, and emotion regulation has made significant theoretical contributions to psychology, behavioral science, and digital addiction studies. We discuss how these implications reify current theories and open new avenues to explain the relationships between these phenomena. Childhood trauma serves as a vulnerability, which, together with modern stressors, such as social media use, strengthens the maladaptive behaviors associated with addiction. These results indicate that digital environments should be recognized as important stressors in psychological models and provide empirical evidence that modern stressors (such as social media) can exacerbate vulnerabilities to psychological distress associated with childhood adversity.

Childhood trauma and social media addiction are also linked through the attachment theory. This theory proposes that social media can allow someone to have their unsatisfied emotional needs satisfied and compensated for in ways that their early relationships are unable to offer, yet this will also lead the individual to act out, but these manifestations of actions are dependent on varying predictions. This is consistent with the self-medication hypothesis, in which people use social media to cope with psychological malaise.

General Strain Theory (GST) suggests that individuals who are stressed or in strain have a higher likelihood of partaking in deviant behaviors, such as addiction, as an alternate response. Combining these theories, social networking attachment can probably be considered a GST derived deviant behavior that pertains to its root and effect in origin by coping against early traumata-related long-life emotional difficulties (Watters et al., 2024).

Emotion regulation, social media addiction, and childhood trauma—implications for specialized treatment in aged-care facilities—Current Research in Geriatrics, Behavioral addictions do not only depend on the addictive properties of the behavior but also on underlying psychological vulnerabilities. It expands the definition of behavioral addiction, implicating developmental influences and affecting dysregulation, and suggests that addictions are a meaning for something else as much as they are responses to stimuli. These theoretical implications provide new perspectives and extend current psychological models, prompting additional studies on the intricate relationship between early life experiences, emotional processes, and contemporary technical environments.

### Practical implications

This finding has practical implications for mental health providers, highlighting the robust relationship between social media addiction, childhood trauma, and difficulties in emotion regulation. Suggestions include the implementation of tailored mental health treatment, digital literacy programs, systematic screening for adversity and emotion regulation in mental health services, special training for professionals, structuring new media with protection measures, public awareness campaigns, school support systems, family interventions, interdisciplinary cooperation, and evidence-based policymaking.

Intervention plans need to be well-informed by the trauma-informed care approach, and emotion regulation skills should be treated to tackle social media addiction from its roots. For clients who require more intensive intervention, reducing the use of social media as a means of coping, evidence-based approaches (e.g., CBT and DBT), and trauma-focused therapies can provide help. Wellbeing is a digital literacy initiative that provides young people with the tools needed to recognize when they are triggered and how dealing with these problems on social media can be harmful, while early identification of signs of addiction can prevent these challenges from starting or worsening.

If a large tenant for social media companies and their stockholders is advertising revenue, it becomes very difficult to get them interested in building ways into social platforms where users can control their use, specifically for people struggling with severe trauma or emotional issues. These platforms could be developed to set usage time limits, remind users to take breaks between gameplays (recommend a regulatory standard), and include mental health resources. Awareness campaigns can make the general population aware of efforts to address mental health needs early in their development as well as advertise services for those suffering from addiction. In particular, supportive school environments may allow students with experience of trauma to develop healthy emotion-regulation skills. Root-family based interventions in the impact of childhood trauma and in still-healthy communication practices, while facilitating healthcare provider/education-tech partnerships, will promote a systems-level approach. Policies aimed at mental health and digital media should consider the implications of research on social media addiction, childhood trauma, and emotional regulation.

## Conclusion

Many types of maltreatment during childhood, including abuse and neglect, have been linked to an increased risk of later life addiction and mental health problems. These traumas can result in deep-ended psychological issues that affect emotional regulation, decision making, and coping skills. Background: Social media addiction is a serious public health concern worldwide and its severity is closely related to childhood maltreatment. People who struggle to regulate their emotions, for example, the inability to control negative affect, might be more prone to engage in behaviors with poor coping mechanisms (such as excessive use of the platform). The relationship between childhood trauma, emotion-regulation problems, and social media addiction is complex. Childhood trauma can skew the way healthy emotion regulation strategies are developed early in life, leading people to resort to maladaptive coping mechanisms such as those associated with addiction. Social media addiction also disrupts emotional regulation, as one grows even less capable of self-validation and immediate gratification. A holistic solution that recognizes core trauma and centers on therapeutic coping mechanisms should be implemented.

### Future directions

A more complete intermediate study is essential to clarify the relationships between childhood trauma, emotional regulation, and social media addiction as well as to improve intervention studies. Future research should further investigate these links. In the present sample, we examined emotion regulation as a mediator of the relationship between childhood trauma and addiction. The impact of culture and social media platforms will be further investigated through cross-cultural studies. Intervention research should focus on improving emotion regulation to decrease the reliance on social media. Additional studies examining neurobiological mechanisms, personality characteristics, family dynamics, and social support are needed. They will also investigate how stress from the COVID-19 pandemic and increased time on social media affect addiction and explore whether there are differences in how girls and boys follow addiction pathways.

## Data Availability

The raw data supporting the conclusions of this article will be made available by the authors without undue reservation.
